# Periodontal Inflamed Surface Area Mediates the Link between Homocysteine and Blood Pressure

**DOI:** 10.3390/biom11060875

**Published:** 2021-06-12

**Authors:** João Botelho, Vanessa Machado, Yago Leira, Luís Proença, José João Mendes

**Affiliations:** 1 Clinical Research Unit (CRU), Egas Moniz Interdisciplinary Research Center, Egas Moniz–Cooperativa de Ensino Superior, 2829-511 Almada, Portugal; vmachado@egasmoniz.edu.pt (V.M.); jmendes@egasmoniz.edu.pt (J.J.M.); 2 Evidence-Based Hub, Egas Moniz Interdisciplinary Research Center, Egas Moniz–Cooperativa de Ensino Superior, 2829-511 Almada, Portugal; lproenca@egasmoniz.edu.pt; 3 Periodontology Unit, UCL Eastman Dental Institute and NIHR UCLH Biomedical Research Centre, University College London, London WC1E 6BT, UK; y.leira@ucl.ac.uk; 4 Periodontology Unit, Faculty of Medicine and Odontology, University of Santiago de Compostela, 15706 Santiago de Compostela, Spain; 5 Medical-Surgical Dentistry (OMEQUI) Research Group, Health Research Institute of Santiago de Compostela (IDIS), 15706 Santiago de Compostela, Spain; 6 Clinical Neurosciences Research Laboratory, Health Research Institute of Santiago de Compostela (IDIS), 15706 Santiago de Compostela, Spain; 7 Quantitative Methods for Health Research (MQIS), Egas Moniz Interdisciplinary Research Center, Egas Moniz–Cooperativa de Ensino Superior, 2829-511 Almada, Portugal

**Keywords:** periodontitis, periodontal disease, periodontal medicine, inflammation and innate immunity, cardiovascular disease(s), oral medicine

## Abstract

Here, we assess the association between homocysteine (Hcy) serum levels and periodontal status in a large representative sample of the National Health and Nutrition Examination Survey (NHANES). Using the 2001–2002 and 2003–2004 NHANES databases, participants with a periodontal examination, medical self-reported data, blood pressure (BP) and blood samples to determine complete blood count, C-reactive protein (CRP) and Hcy levels. We then calculated the periodontal inflamed surface area (PISA) and the periodontal epithelial surface area (PESA). Multivariable regression analysis explored the association between Hcy, periodontal measures and BP. Mediation analysis was performed to understand the effect of PISA and PESA in the link between Hcy and BP. 4021 participants fulfilled the inclusion criteria. Hcy levels showed significant correlations with systolic BP, diastolic BP, PISA, PESA and age. PESA showed to be significantly associated with Hcy both for the crude and adjusted models (*p* < 0.01), but not PISA (*p* > 0.05). In the association of Hcy with systolic BP, PISA significantly mediated 17.4% and PESA 0.9%. In the association of Hcy with diastolic BP, PISA significantly mediated 16.3% and PESA 47.2%. In conclusion, Hcy and periodontitis are associated. Further, both PISA and PESA significantly mediated the association of Hcy with systolic BP and diastolic BP. Future studies shall deepen the mechanisms by which Hcy levels increase in a clinical situation of periodontitis.

## 1. Introduction

Homocysteine (Hcy) is an amino acid synthesized as an intermediate metabolite from the methionine (Met) biosynthesis [1,2]. In plasma, 1% of Hcy circulates in its free form while the remaining 99% is bound to proteins [3], and its concentration normally ranges between 5–15 µmol/L [2]. In cases of hyperhomocysteinemia (defined as total Hcy in plasma >15 µmol/L) [4,5], the main causes may be impaired metabolism of Met or defective cofactors in the remethylation to methionine pathway (involving B12 and folic acid) or the trans-sulfuration pathway (involving B6) [6].

Hyperhomocysteinemia has been implicated with high blood pressure (BP) [7] as the increase in Hcy levels inhibits the synthesis of endothelial nitric oxide, causing its dysfunction and damages the myocardium via the excessive production of reactive oxygen radicals [8,9]. Hcy was firstly introduced as a candidate in the link of periodontal and cardiovascular conditions [10], but ever since, studies on this topic have been relatively scarce.

Periodontitis is a chronic inflammatory condition of the periodontium driven by a dysbiotic plaque [11,12]. Hcy was firstly reported to be associated with periodontitis [13,14], and was suggested as an inflammatory marker of periodontitis as its levels decrease after periodontal treatment [15]. Moreover, periodontitis plays a very active role in cardiovascular illnesses whether through systemic inflammation affecting endothelial function and through vascular dysfunction caused by bacteremia and cell infiltrate secondary to periodontal microbiota [16]. Thus, a possible form of action of periodontitis emerges in the Hcy-hypertension link (i.e., mediation effect), however this has never been studied.

The primary aim of the present study was to assess the association between Hcy serum levels and periodontal parameters in a large representative sample of the National Health and Nutrition Examination Survey (NHANES). As a secondary aim, we appraised the mediation effect of periodontal measurements in the link between Hcy and BP.

## 2. Materials and Methods

### 2.1. Study Design and Participants

In this study, we performed a secondary analysis using datasets from the NHANES of 2001–2002 and 2003–2004 [17]. Data was gathered through interviews, physical and laboratory exams, and both waves have been reviewed and approved by the Centers for Disease Control (CDC) and Prevention National Center for Health Statistics Research (NCHS) Ethics Review Board. All included participants provided a written informed consent.

For the purpose of this study, the following inclusion criteria were defined: participants with 18 years old or older, undergone periodontal examination and had completed measurement of plasma Hcy. This study follows the strengthening the reporting of observational studies in epidemiology (STROBE) guideline [18] (Table S1).

### 2.2. Periodontal Examination

The periodontal examination was carried out using a partial-mouth protocol using randomly assigned quadrants (one on the upper arch, and one on the lower arch), by calibrated examiners as previously defined [19]. Three periodontal measures were registered: periodontal pocket depth (PPD), clinical attachment loss (CAL) and bleeding on probing (BOP). These measures were made at three buccal sites per tooth (mesio-, mid- and disto-buccal). The presence of periodontitis was diagnosed according to the CDC/American Academy of Periodontology (AAP) case definition [20]. Then, periodontitis was categorized as mild (≥2 interproximal sites with CAL ≥ 3 mm; and ≥2 interproximal sites with PPD ≥ 4 mm (not on the same tooth) or 1 site with PPD ≥ 5 mm), moderate (≥2 interproximal sites with CAL ≥ 4 mm [not on the same tooth] or ≥2 interproximal sites with PPD ≥ 5 mm, also not on the same tooth) and severe (≥2 interproximal sites with CAL ≥ 6 mm (not on the same tooth) and ≥1 interproximal site with PPD ≥ 5 mm). The overall number of participants with periodontitis was the sum of mild, moderate and severe periodontitis.

Next, we computed for each tooth the PISA and the PESA for each specific participant. PISA and PESA were used as continuous variables. PISA comprised the surface area of bleeding pocket epithelium and PESA is the root surface area of that tooth, which is covered with pocket epithelium [21,22]. PISA and PESA were calculated using a Microsoft Excel spreadsheet, in the following steps:

- Calculation of mean CAL and gingival recession for each particular tooth;
- Linear mean CAL and gingival recession is used to calculate PESA;
- For each tooth, PISA is calculated through the multiplication of PESA by the proportion of sites around the tooth with BOP;
- The sum of all individual PISA and PESA scores, in mm^2^, provides an overall area, respectively, for each participant.

### 2.3. Plasma Homocysteine Measurement

For NHANES 2001, total Hcy in plasma was quantified by a fully automated fluorescence polarization immunoassay (FPIA) (Abbott Hcy IMX (HCY) assay, Abbott Laboratories, Chicago, IL, USA) [23]. For NHANES 2002 and 2003–2004, total Hcy in plasma was measured by other FPIA (Abbott Diagnostics (Abbott AxSym analyzer, Abbott^®^), Abbott Laboratories, Chicago, IL, USA) [23,24,25]. Both methods employed the same reagent kit. Moreover, both approaches presented equivalence as the FPIA results were successfully compared to high performance liquid chromatography with fluorometric detection at 385 nm excitation and 515 nm emission [23,25]. Hyperhomocysteinemia was defined as total Hcy in plasma >15 µmol/L [4,5].

In both FPIA approaches [23,24,25], dithiothreitol (DTT) was used to free thiol, then S-adenosyl-Hcy (SAH) hydrolase was applied to catalyze the conversion of Hcy to SAH in the presence of added adenosine. Then, FPIA was performed using a specific monoclonal antibody and fluoresceinated SAH analog tracer [24]. In the first FPIA method, total Hcy concentrations were calculated by the Abbott IMx^®^ (Illinois, USA) using a machine-stored calibration curve, while in the second was calculated by the Abbott Axsym^®^ (Illinois, USA) using a machine-stored calibration curve [26,27].

### 2.4. Covariates

We included sociodemographic data, including age, gender, race/ethnicity (categorized as mexican american, non-hispanics white, non-hispanics black, other hispanics and other races), education level (categorized as less than high school, complete high school or similar, higher than high school) and the poverty income ratio.

Smoking status was categorized as never (<100 cigarettes smoked in life and not currently smoking), former (≥100 cigarettes in life and not currently smoking) and active smoker (≥100 cigarettes in life and currently smoking). We calculated BMI as weight in kilograms (kg) divided by height in meters squared (m^2^).

For BP measurement, sitting SBP and DBP were determined by trained and calibrated examiners. Both BP measures resulted from the average of three consecutive measurements separated by a 5-min interval following a standardized protocol [28]. Average measurements of SBP and DBP were obtained from three consecutive readings. Hypertension was defined as values of SBP ≥ 140 mmHg or DBP ≥ 90 mmHg or the use of antihypertensive medication [29,30].

Blood levels data included WBC Count (10^9^/L), segmented neutrophils (10^9^/L), hemoglobin A1c (HbA1c) (%), CRP (mg/dL), vitamin B12 (pg/mL) and folate (ng/mL). Vitamin B12 and folate were considered given its link to the Hcy biosynthesis [1,2].

### 2.5. Data Management, Test Methods and Analysis

Databases from the NHANES 2001–2002 and 2003–2004 were uploaded and treated with R (version for Macintosh). For the purpose of periodontal diagnosis, data was exported to a spreadsheet processor with algorithms, as previously described [31], to compute the periodontal status, PISA and PESA (as in Nesse et al. [22]). After checking for data normality and homoscedasticity, measures were reported through mean (standard deviation (SD)) for continuous variables, and a number of cases (n) and percentage (%) for categorical variables. *T*-test was used to compare continuous measures and chi-square test to compare categorical variables according to the periodontal status (periodontitis vs. no periodontitis).

Pearson coefficient was used to assess the correlation between the Hcy and CRP with age, BMI, periodontal clinical measures (PISA and PESA), WBC, segmented neutrophils, vitamin B12 and folate. A multivariate stepwise adjusted linear regression was used to model the influence of Hcy on PISA and PESA. Model variables were selected among significant variables according to the periodontal status (Table 1). An initial crude model (Model 1), followed by seven progressively adjusted models were generated (Model 2—Includes adjustment for Hcy and age; Model 3—Includes adjustment for Hcy, age and BMI; Model 4—Includes adjustment for Hcy, age, BMI and SBP; Model 5—Includes adjustment for Hcy, age, BMI, SBP and WBC; Model 6—Includes adjustment for Hcy, age, BMI, SBP, WBC and vitamin B12; Model 7—Includes adjustment for Hcy, age, BMI, SBP, WBC, vitamin B12 and folate; Model 8—Includes adjustment for Hcy, age, BMI, SBP, WBC, vitamin B12, folate and HbA1c). Multivariate stepwise adjusted linear regressions were carried out on the association of SBP and DBP with PISA or PESA (Table S2), and homocysteine levels and SBP or DBP (Table S3).

The mediating effect of PISA and PESA in the association of Hcy with SBP and DBP was carried out using the R package ‘lavaan’. Mediation analysis was done through the establishment of three pathways (a, b and c) (Figure S1). Total effect (c-path) evaluated the relationship between the exposure (Hcy) and the outcomes of interest (SBP or DBP). The a-path assessed the direct effect of the exposure (Hcy) on the mediators of interest (PISA or PESA). The b-path measured the mediators (PISA or PESA) direct effect on the outcomes of interest (SBP or DBP). The mediation effect was computed through multiplying a-path with b-path. The proportion of the mediated effect was calculated using the following formula: (total effect–direct effect) × 100. All mediation models have been adjusted for sociodemographic variables (age, gender, race, education), smoking habit, BMI, systemic status (number of chronic medical conditions, hypertension, diabetes mellitus, Hba1c), vitamin B12 and folate. A value of *p* < 0.05 was considered significant.

## 3. Results

### 3.1. Characteristics of the Study Sample

With a primary sample of 21,161 participants, 17,140 were excluded based on the defined the inclusion criteria, thus, resulting in a final sample of 4021 patients (Figure S2). The overall characteristics of these participants are shown in Table 1. The mean age of the participants was 41.7 (±18.9) years, with balanced proportion between males and females. Participants were mostly non-hispanic black participants (48.4%), higher education level (47.4%) and self-reported as never smoker (59.1%). Moreover, this sample had an average body mass index (BMI) of 27.9 (±7.1) kg/m^2^, indicating an average overweight population. A total of 734 participants were diagnosed as periodontitis cases (18.3%). Periodontitis cases presented significantly differences regarding mean age, gender, race/ethnicity, education level, smoking status, family poverty ratio, Hcy mean levels, Hcy category levels, chronic medical conditions, diabetes mellitus, hypertension, BP (systolic BP (SBP) and diastolic BP (DBP)), C-reactive protein (CRP), periodontal inflamed surface area (PISA) and periodontal epithelial surface area (PESA) (*p* < 0.001). Mean BMI (*p* = 0.011) and folate (*p* = 0.040) were also different among periodontal groups, however, vitamin B12 was not (*p* = 0.880).

### 3.2. Correlation Estimates of Hcy Compared to CRP

**Figure 1:**
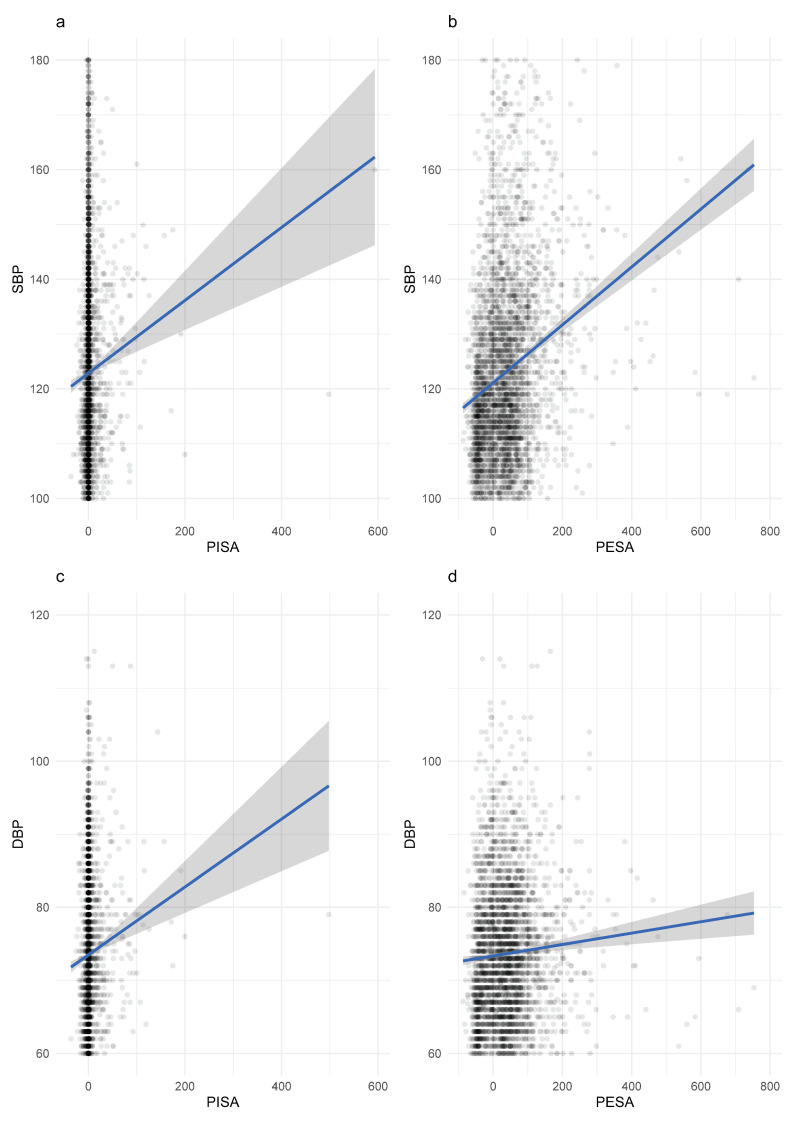
Scatterplots displaying the correlation between PISA with SBP (**a**) and DBP (**c**), and PESA with SBP (**b**) and DBP (**d**).

To understand if Hcy and CRP displayed alike behavior with similar variables, we investigated the correlation of Homocysteine and CRP with relevant continuous variables (Table 2). In the Hcy levels, age had a moderate correlation effect (*p* < 0.001), while SBP, DBP, PISA and PESA had a mild correlation effect (*p* < 0.001). Furthermore, we confirmed the association of Hcy levels with vitamin B12 and folate (*p* < 0.001). In the overall sample, CRP had a significantly mild correlation with BMI (*p* < 0.001), white blood cells (WBC) counts (*p* < 0.001), segmented neutrophils (*p* < 0.001) and age (*p* < 0.05). The correlations between PISA and PESA with SBP and DBP were graphically displayed (*p* < 0.01) (Figure 1).

Next, we examined the linear relationship of Hcy with PISA and PESA in crude (Model 1) and adjusted models (Models 2–8) (Table 3). Linear regression models confirmed that PISA is significantly associated with Hcy (*p* < 0.001) however, that not occurred in the adjusted models. While for PESA, both for the crude and adjusted models, it was significantly associated with Hcy (*p* < 0.01). The linear association of SBP and DBP with PISA and PESA was also carried out (Table S1), with DBP being associated with PISA in all adjusted models. The linear association of Hcy with SBP and DBP was found to be significant in all adjusted models (Table S2).

### 3.3. Mediation Analyses

There was evidence that the association between Hcy with BP (SBP and DBP) were mediated by PISA and PESA. Mediation analyses demonstrated that PISA was estimated to mediate 17.39% and 16.33% of the total association between Hcy with SBP and DBP, respectively (*p* < 0.001) (Table 4). Furthermore, PESA was estimated to mediate 0.89% and 47.20% of the total association between Hcy with SBP and DBP, respectively (*p* < 0.001) (Table 4 and Table 5).

## 4. Discussion

In this exploratory study, we found periodontitis cases to present higher Hcy levels, and this association showed significance even when adjusted for multiple confounding variables. Moreover, two periodontal parameters (PISA and PESA) demonstrated to have potential mediating effect in the link between Hcy and BP. These findings may support an association between Hcy and periodontitis.

Overall, these results, in our view, are novel because they suggest that such association may rely more on the destruction of periodontal tissues (PESA) rather than inflamed surface area (PISA). That is to say, PESA as a reflection of the subgingival space harboring bacteria underlines the greater importance of the area of bacterial colonization and its increase during periodontal destruction in this association. The bacterial role related to methionine pathways is based on the production of volatile sulfur compounds by periodontal bacteria, causing the commonly detected malodor in periodontal patients [32,33,34]. At this stage, these results only highlight a possible connection point, and the reader must bear in mind that they are solely hypothetical. Though, the invasion of periodontal bacteria through the ulcerated epithelium into the blood stream has been shown, with eventual hostile systemic consequences [35,36].

In fact, Hcy was initially proposed in periodontal medicine as a candidate in the link of periodontal with cardiovascular conditions [10]. Comprehensively, Hcy inhibits nitric oxide in the endothelium affecting its vasodilation function and leads to excessive reactive oxygen radicals in the myocardium [8,9]. The elevated serum levels of Hcy result, therefore, in a higher risk for endothelial cell injury by inducing apoptotic cell death of endothelial cells and smooth muscle cells [37,38], and is collectively seen as an inflammatory reaction in the blood vessels [39]. Furthermore, periodontitis was proposed to affect vascular function through boosted systemic inflammation and bacteremia, instigating vascular damage (by increased cytokines, immune cells, nitric oxide and reactive oxygen species) [16]. Such as Hcy, periodontal bacteria have been shown to decrease the bioavailability of nitric oxide [40] and this biological pathway may be a starting point in this association.

In this sense and given the cross-sectional nature of this study (also discussed later), we cannot discern whether this is a unilateral (and triggered by Hcy or periodontitis) or bilateral nature. On the one hand, untreated periodontitis further inflammatory burden that has been shown to increase the risk towards cardiovascular disease [41]. On the other hand, indirect evidence from a clinical trial demonstrated that adjunctive folate during periodontal treatment reduced plasma Hcy, yet with unknown biological reasons and long-term clinical consequences (i.e., reduced risk of high BP) [42].

Another important mechanistic nexus can be oxidative stress, which is related to both hyperhomocysteinemia [43] and periodontitis [44]. In both circumstances, reactive oxygen species are overproduced and cause unbalanced milieus causing tissue damage [43,44]. Nevertheless, we were not able to explore further this as the NHANES does not provide suitable variables representing oxidative stress, and this is a matter to address in the future.

### Strengths and Limitations

The present cross-sectional study has specific fallouts worth examining. The observational nature of NHANES precluded any inference of causality or temporal link. Furthermore, information regarding systemic inflammation was limited as CRP levels were the only recognized and accepted hallmark available, thus limiting the validity of these results. For this reason, data on other important markers of inflammation, such as cytokines (Tumor Necrosis Factor-α and interleukins) might be of interest to study in future studies.

When exploring the association and mediation effect, these results should be interpreted cautiously. However, previous studies have contributed to support a conceivable causal link between Hcy and periodontitis as studies have found higher levels of Hcy in periodontitis cases [13,14,15,42] and a randomized intervention trial demonstrated the effectiveness of periodontal treatment in reducing Hcy levels [42]. Although, studies with small samples have reported this association [13,14], our study may stand-out from the others by the considerable increase in participants. Furthermore, this sample is based on two consecutive NHANES waves, and are representative of the American population. The number of covariates included in our analyses were comprehensive.

The periodontal assessment was made by calibrated examiners yet a partial-mouth protocol has been taken place, which may contribute to less accurate and precise results [31,45]. Despite this, our estimates revealed very interesting values of both correlation and mediation.

## 5. Conclusions

Hcy serum levels are associated with the periodontal status. Hcy levels demonstrated more relationship with PISA and PESA, BP and age. Compared to CRP, Hcy showed different associations. Besides, PISA and PESA might play a mediation effect on the link between Hcy and BP.

## Figures and Tables

**Table 1:** Sample characteristics according to periodontal status (*n* = 4021).

Variable	No Periodontitis (*n* = 3287)	Periodontitis (*n* = 734)	*p*-Value	Overall (*n* = 4021)
Age (years), mean (SD)	38.4 (17.8)	56.8 (15.8)	<0.001	41.7 (18.9)
Gender, % (*n*)				
Males	46.2 (1520)	61.6 (452)	<0.001	49.0 (1972)
Females	53.8 (1767)	38.4 (282)		51.0 (2049)
Race/ethnicity, % (*n*)				
Mexican American	21.0 (690)	26.0 (191)	0.001	21.9 (881)
Non-Hispanic White	3.4 (111)	4.0 (29)		3.5 (140)
Non-Hispanic Black	50.0 (1643)	41.6 (305)		48.4 (1948)
Other Hispanic	21.8 (715)	24.0 (176)		22.2 (891)
Other races	3.9 (128)	4.5 (33)		4.0 (161)
Education level, % (*n*)				
<High school	24.2 (797)	37.9 (278)	<0.001	26.7 (1075)
High school	25.6 (842)	26.8 (197)		25.8 (1039)
>High school	50.1 (1647)	35.1 (258)		47.4 (1905)
Smoking status, % (*n*)				
Never	63.9 (2100)	37.5 (275)	<0.001	59.1 (2375)
Former	18.3 (603)	31.5 (231)		20.7 (834)
Current	17.8 (584)	31.1 (228)		20.2 (812)
BMI (kg/m^2^), mean (SD)	27.7 (7.0)	28.5 (7.2)	0.011	27.9 (7.1)
Hcy (µmol/L), mean (SD)	8.2 (3.9)	9.8 (4.0)	<0.001	8.5 (4.0)
Hcy elevated level category, % (*n*)	2.8 (92)	6.1 (45)	<0.001	3.4 (137)
CRP (mg/dL), mean (SD)	0.4 (0.8)	0.5 (0.8)	<0.001	0.4 (0.8)
Chronic medical conditions, mean (SD)	0.8 (1.5)	1.4 (2.7)	<0.001	0.9 (1.8)
Diabetes Mellitus, *n* (%)	5.0 (164)	14.7 (108)	<0.001	6.8 (272)
Hypertension, *n* (%)	12.1 (399)	29.4 (216)	<0.001	15.3 (615)
Mean SBP (mmHg), mean (SD)	120.0 (17.6)	132.0 (21.2)	<0.001	122.2 (18.9)
Mean DBP (mmHg), mean (SD)	69.4 (11.7)	72.0 (11.9)	<0.001	69.8 (11.8)
PISA (mm^2^), mean (SD)	0.8 (7.9)	13.5 (38.4)	<0.001	3.1 (18.5)
PESA (mm^2^), mean (SD)	17.3 (51.4)	118.0 (107.0)	<0.001	35.6 (75.7)
Vitamin B12 (pg/mL), mean (SD)	550.0 (1120.0)	554.0 (521.0)	0.880	550.9 (1037.1)
Folate (ng/mL), mean (SD)	12.6 (7.8)	14.6 (26.3)	0.040	12.9 (13.3)

<—less; >—higher; BMI—Body Mass Index; CRP—C-Reactive Protein; DBP—Diastolic Blood Pressure; Hcy—Homocysteine; n—number of cases; PESA—Periodontal Epithelial Surface Area; PISA—Periodontal Inflamed Surface Area; SBP—Systolic Blood Pressure; SD—Standard Deviation.

**Table 2:** Correlation of CRP and Hcy with sociodemographic and clinical variables (*n* = 4021).

Variable	CRP (mg/dL)	Homocysteine (µmol/L)
Age (years)	0.036 *	0.326 **
Hcy (µmol/L)	−0.019	-
BMI (g/m^2^)	0.215 **	0.003
SBP (mmHg)	0.037 *	0.274 **
DBP (mmHg)	0.015	0.124 **
PISA (mm^2^)	0.030	0.054 **
PESA (mm^2^)	0.031	0.177 **
WBC (10^9^/L)	0.154 **	−0.026
Segmented Neutrophils (10^9^/L)	0.143 **	−0.035 *
Vitamin B12 (pg/mL)	0.006	−0.058 **
Folate (ng/mL)	−0.011	−0.045 **

Pearson correlation, * *p* < 0.05, ** *p* < 0.001. BMI—Body Mass Index; CRP—C-reactive Protein; DBP—Diastolic Blood Pressure; Hcy—Homocysteine; PISA—Periodontal Inflamed Surface Area; PESA—Periodontal Epithelial Surface Area; SBP—Systolic Blood Pressure; WBC—White blood cells counts.

**Table 3:** Crude and adjusted linear regression models of homocysteine levels and PISA or PESA for the overall sample with the respective B coefficient and standard error (SE) (*n* = 4021).

Model	Homocysteine	
	PISA	PESA
1	0.012 ** (0.003)	0.009 ** (0.001)
2	0.005 (0.003)	0.002 * (0.001)
3	0.005 (0.003)	0.002 * (0.001)
4	0.004 (0.003)	0.002 * (0.001)
5	0.004 (0.003)	0.002 * (0.001)
6	0.004 (0.003)	0.002 * (0.001)
7	0.003 (0.003)	0.002 * (0.001)
8	0.003 (0.003)	0.002 * (0.001)

Values are presented as B coefficient (SE). PISA—Periodontal Inflamed Surface Area; PESA—Periodontal Epithelial Surface Area. Model 1—Unadjusted model for Hcy; Model 2—Includes adjustment for Hcy and age; Model 3—Includes adjustment for Hcy, age and BMI; Model 4—Includes adjustment for Hcy, age, BMI and SBP; Model 5—Includes adjustment for Hcy, age, BMI, SBP and WBC; Model 6—Includes adjustment for Hcy, age, BMI, SBP, WBC and vitamin B12; Model 7—Includes adjustment for Hcy, age, BMI, SBP, WBC, vitamin B12 and folate; Model 8—Includes adjustment for Hcy, age, BMI, SBP, WBC, vitamin B12, folate and HbA1c (%). * *p* < 0.01; ** *p* < 0.001.

**Table 4:** Mediation analysis of the effects of PISA and PESA on the association of Hcy levels with SBP (*n* = 4021).

**Exposure: Hcy/Outcome: SBP Mediator: PISA**			
**Effect**	**Estimate**	**SE**	***p*-value**
a (exposure → mediator)	0.156	0.076	0.041
b (mediator → outcome)	0.076	0.017	<0.001
c (total effect)	0.069	0.006	<0.001
c′ (direct effect)	0.012	0.003	<0.001
ab (mediated effect)	0.012	0.006	<0.001
ab/c (PISA percentage mediated) = 17.4%			
**Exposure: Hcy/Outcome: SBP Mediator: PESA**			
**Effect**	**Estimate**	**SE**	***p*-value**
a (exposure → mediator)	0.006	0.001	<0.001
b (mediator → outcome)	1.176	0.074	<0.001
c (total effect)	0.900	0.063	<0.001
c′ (direct effect)	0.892	0.063	<0.001
ab (mediated effect)	0.008	0.001	<0.001
ab/c (PISA percentage mediated) = 0.9%			

PISA—Periodontal Inflamed Surface Area; PESA—Periodontal Epithelial Surface Area; SBP—Systolic Blood Pressure. Models adjusted for sociodemographic variables (age, gender, race, education), smoking habit, BMI, systemic status (number of chronic medical conditions, diabetes mellitus, hemoglobin A1c [HbA1c]), vitamin B12 and folate. Abbreviations: Hcy—homocysteine; PISA—Periodontal Inflamed Surface Area; PESA—Periodontal Epithelial Surface Area; SBP—Systolic Blood Pressure.

**Table 5:** Mediation analysis of the effects of PISA and PESA on the association of Hcy levels with DBP (*n* = 4021).

**Exposure: Hcy/Outcome: DBP Mediator: PISA**			
**Effect**	**Estimate**	**SE**	***p*-value**
a (exposure → mediator)	0.219	0.074	0.003
b (mediator → outcome)	0.035	0.010	0.001
c (total effect)	0.049	0.006	<0.001
c′ (direct effect)	0.041	0.005	<0.001
ab (mediated effect)	0.008	0.003	0.017
ab/c (PISA percentage mediated) = 16.3%			
**Exposure: Hcy/Outcome: DBP Mediator: PESA**			
**Effect**	**Estimate**	**SE**	***p*-value**
a (exposure → mediator)	3.222	0.297	<0.001
b (mediator → outcome)	0.011	0.002	<0.001
c (total effect)	0.072	0.009	<0.001
c′ (direct effect)	0.038	0.005	<0.001
ab (mediated effect)	0.034	0.008	<0.001
ab/c (PISA percentage mediated) = 47.2%			

PISA—Periodontal Inflamed Surface Area; PESA—Periodontal Epithelial Surface Area; DBP—Diastolic Blood Pressure. Models adjusted for sociodemographic variables (age, gender, race, education), smoking habit, BMI, systemic status (number of chronic medical conditions, diabetes mellitus, hemoglobin A1c [HbA1c]), vitamin B12 and folate. Abbreviations: Hcy—homocysteine; PISA—Periodontal Inflamed Surface Area; PESA—Periodontal Epithelial Surface Area; DBP—Diastolic Blood Pressure.

## Data Availability

Data will be provided upon reasonable request.

## Supplementary Materials

The following are available online at https://www.mdpi.com/article/10.3390/biom11060875/s1, Figure S1: Participant’s flowchart, Figure S2: Path diagram of the mediation analysis models, Table S1: Crude and adjusted linear regression models of SBP and DBP with PISA or PESA for the overall sample with the respective B coefficient and standard error (SE) (*n* = 4021), Table S2: Crude and adjusted linear regression models of homocysteine levels and SBP or DBP for the overall sample with the respective B coefficient and standard error (SE) (*n* = 4021), Table S3: Strobe statement—Checklist of items that should be included in reports of case-control studies.
