# Supplementary material for: Periodontal Inflamed Surface Area Mediates the Link between Homocysteine and Blood Pressure
Source: Biomolecules. 2021 Jun 12;11(6):875. doi: 10.3390/biom11060875 (PMC8231519; doi:10.3390/biom11060875)
Supplement: Supplementary file 1 [file biomolecules-11-00875-s001.zip › Final Table S2.pdf]

Appendix S2. Crude and adjusted linear regression models of SBP and DBP with PISA or PESA for the overall sample with the respective B coefficient and standard error (SE) (n=4,021).

	SBP		DBP	
Model	PISA	PESA	PISA	PESA
1	0.085*** (0.016)	0.063 (0.003)***	0.039 (0.010)***	0.014 (0.002)***
2	0.028* (0.013)	-0.001 (0.004)	0.024 (0.010)*	-0.003 (0.003)
3	0.025 (0.013)	-0.001 (0.004)	0.022 (0.010)*	-0.002 (0.003)
4	0.023 (0.013)	-0.001 (0.004)	0.021 (0.010)*	-0.002 (0.003)
5	0.023 (0.013)	-0.001 (0.004)	0.022 (0.010)*	-0.002 (0.003)
6	0.023 (0.013)	-0.001 (0.004)	0.022 (0.010)*	-0.002 (0.003)
7	0.023 (0.013)	-0.001 (0.004)	0.022 (0.010)*	-0.002 (0.003)
8	0.023 (0.013)	-0.001 (0.004)	0.022 (0.010)*	-0.002 (0.003)

Values are presented as B coefficient (SE).

Model 1 - Unadjusted model; Model 2 - Includes adjustment for age; Model 3 - Includes adjustment for age and BMI; Model 4 - Includes adjustment for age, BMI and Homocysteine; Model 5 - Includes adjustment for age, BMI, Homocysteine and WBC; Model 6 - Includes adjustment for age, BMI, Homocysteine, WBC and Vitamin B12; Model 7 - Includes adjustment for age, BMI, Homocysteine, WBC, Vitamin B12 and Folate; Model 8 - Includes adjustment for age, BMI, Homocysteine, WBC, Vitamin B12, Folate and HbA1c (%). * $p < 0.05$; ** $p < 0.01$; *** $p < 0.001$.
