# Supplementary material for: Periodontal Inflamed Surface Area Mediates the Link between Homocysteine and Blood Pressure
Source: Biomolecules. 2021 Jun 12;11(6):875. doi: 10.3390/biom11060875 (PMC8231519; doi:10.3390/biom11060875)
Supplement: Supplementary file 1 [file biomolecules-11-00875-s001.zip › Final Table S3.pdf]

Appendix S3. Crude and adjusted linear regression models of homocysteine levels and SBP or DBP for the overall sample with the respective B coefficient and standard error (SE) (n=4,021).

	Homocysteine	
Model	SBP	DBP
1	0.058*** (0.003)	0.042*** (0.005)
2	0.028*** (0.004)	0.018*** (0.005)
3	0.029*** (0.004)	0.020*** (0.005)
4	0.029*** (0.004)	0.019*** (0.005)
5	0.029*** (0.004)	0.020*** (0.005)
6	0.029*** (0.004)	0.020*** (0.005)
7	0.029*** (0.004)	0.020*** (0.005)
8	0.029*** (0.004)	0.019*** (0.005)
9	0.029*** (0.004)	0.019*** (0.005)

Values are presented as B coefficient (SE).

Model 1 - Unadjusted model; Model 2 - Includes adjustment for age; Model 3 - Includes adjustment for age and BMI; Model 4 - Includes adjustment for age, BMI and PISA; Model 5 - Includes adjustment for age, BMI, PISA and PESA; Model 6 - Includes adjustment for age, BMI, PISA, PESA and WBC; Model 7 - Includes adjustment for age, BMI, PISA, PESA, WBC, and Vitamin B12; Model 8 - Includes adjustment for age, BMI, PISA, PESA, WBC, Vitamin B12 and Folate; Model 9 - Includes adjustment for age, BMI, PISA, PESA, WBC, Vitamin B12, Folate and HbA1c (%). * $p < 0.05$; ** $p < 0.01$; *** $p < 0.001$.
