# Supplementary figures and images for: Periodontal Inflamed Surface Area Mediates the Link between Homocysteine and Blood Pressure
Source: Biomolecules. 2021 Jun 12;11(6):875. doi: 10.3390/biom11060875 (PMC8231519; doi:10.3390/biom11060875)

**Appendix S5.** Path diagram of the mediation analysis models.

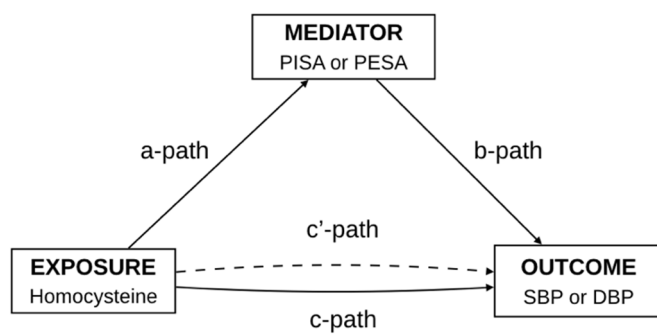

Supplement: Supplementary file 1 [file biomolecules-11-00875-s001.zip › Final Figure S1.pdf]

**Appendix S1.** Flowchart of participants.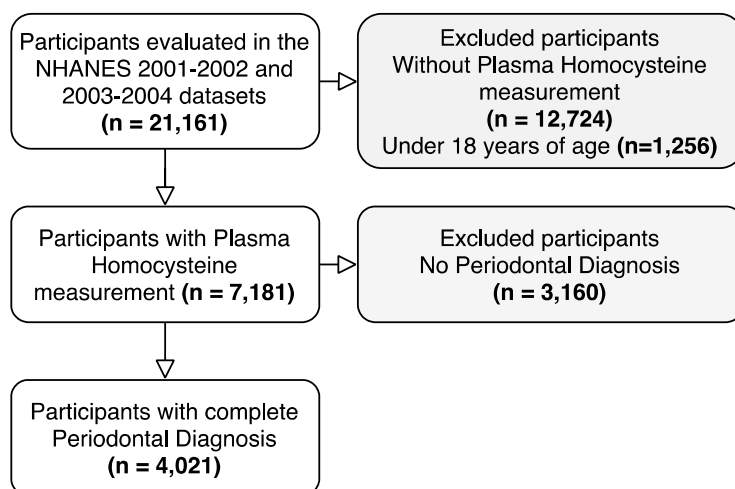

Supplement: Supplementary file 1 [file biomolecules-11-00875-s001.zip › Final Figure S2.pdf]
